# The High‐Sensitivity HEART Pathway Safely Reduces Hospitalizations Regardless of Sex or Race in a Multisite Prospective US Cohort

**DOI:** 10.1002/clc.70027

**Published:** 2024-10-17

**Authors:** Campbell J. Veasey, Anna C. Snavely, Zechariah L. Kearns, Nicklaus P. Ashburn, Tara Hashemian, Simon A. Mahler

**Affiliations:** ^1^ Department of Emergency Medicine Wake Forest University School of Medicine (WFUSOM) Winston‐Salem North Carolina USA; ^2^ Department of Biostatistics and Data Science WFUSOM Winston‐Salem North Carolina USA; ^3^ Department of Implementation Science WFUSOM Winston‐Salem North Carolina USA; ^4^ Department of Epidemiology and Prevention WFUSOM Winston‐Salem North Carolina USA

**Keywords:** chest pain, disparities, HEART pathway, high‐sensitivity cardiac troponin, race, risk stratification, sex

## Abstract

**Background:**

The high‐sensitivity HEART pathway (hs‐HP) risk stratifies emergency department (ED) patients with chest pain. It is unknown if its safety and effectiveness vary by sex or race.

**Methods:**

We conducted a subgroup analysis of the hs‐HP implementation study, a pre−post interrupted time series at five US EDs. The pre‐implementation period (January 2019 to April 2020) utilized the traditional HEART pathway with contemporary troponin (Siemens) and the post‐implementation period (November 2020 to February 2022) used the hs‐HP using hs‐cTnI (Beckman Coulter). Patients were risk‐stratified using the hs‐HP to rule‐out, observation, and rule‐in groups. Safety and effectiveness outcomes were 30‐day all‐cause mortality or myocardial infarction (MI) and 30‐day hospitalization.

**Results:**

Twenty‐six thousand and one hundred twenty‐six patients were accrued (12 317 pre‐ and 13 809 post‐implementation), of which 35.3% were non‐White and 52.7% were female. Among 9703 patients with complete hs‐HP assessments, 48.6% of White and 55.4% of non‐White patients were ruled‐out (*p* < 0.001). Additionally, 47.3% of males and 54.4% of females were ruled‐out (*p* < 0.001). Among rule‐out patients, 0.3% of White versus 0.3% of non‐White patients (*p* = 0.98) and 0.3% of females versus males 0.3% (*p* = 0.90) experienced 30‐day death or MI. Post‐implementation, 30‐day hospitalization decreased 17.2% among White patients (aOR 0.49, 95% CI: 0.45−0.52), 14.1% among non‐White patients (aOR 0.53, 95% CI: 0.48−0.59), 15.6% among females (aOR 0.50, 95% CI: 0.46−0.54), and 16.6% among males (aOR 0.51, 95% CI: 0.47−0.56). The interactions for 30‐day hospitalization between hs‐HP implementation and race (*p* = 0.10) and sex (*p* = 0.69) were not significant.

**Conclusions:**

The hs‐HP safely decreases 30‐day hospitalizations regardless of sex or race. However, it classifies more non‐White patients and women to the rule‐out group.

## 1 Introduction

Acute chest pain is a common emergency department (ED) complaint, accounting for up to 6.5 million visits per year [1]. To evaluate for acute coronary syndrome (ACS), guidelines recommend using accelerated diagnostic protocols (ADPs), like the history, electrocardiogram (ECG), age, risk factors, and troponin (HEART) pathway, a validated ADP that combines a patient's HEAR score with serial troponin measures [2, 3]. However, the original HEART pathway was designed and validated using contemporary troponin assays and differs from ADPs derived using high‐sensitivity cardiac troponin (hs‐cTn) measures, such as the 0/1 h algorithm, because it does not incorporate very low initial measures or a combination of initial cut points and delta values to exclude myocardial infarction (MI) [4]. To address these shortcomings, our team recently validated a modified high‐sensitivity HEART pathway (hs‐HP) that follows a structure similar to the original HEART pathway but utilizes a HEAR score with 0‐ and 2‐h hs‐cTnI measures with delta values or a single low hs‐cTnI measure (below the limit of quantification) to risk stratify patients into rule‐out, observation, or rule‐in zones [5]. However, the safety and effectiveness of this hs‐HP have not yet been studied among sex and race subgroups.

There are known differences in cardiovascular outcomes based on sex and race. ED clinicians often have lower risk estimates and order less testing among women and non‐White patients with chest pain [6, 7, 8, 9, 10, 11]. Yet, non‐White patients are 30% more likely to die of heart disease than White patients [12] and women with ACS have higher mortality rates compared to age‐matched men [13, 14]. Non‐White and female patients with ACS are less likely to be hospitalized than their White and male counterparts [15]. Additionally, non‐White patients are less likely to receive objective cardiac testing (OCT: stress testing, coronary computed tomography angiography [CCTA], and invasive angiography) [15, 16]. Given these cardiovascular care disparities in female and non‐White patients, it is important to determine if hs‐HP performance varies by sex or race [17].

This is the first study to evaluate the safety and effectiveness of the hs‐HP among men versus women and White versus non‐White patients presenting to the ED with chest pain. The objectives were to determine and compare among these key subgroups the (1) diagnostic performance of the hs‐HP for 30‐day all‐cause death or MI as well as (2) hospitalization rates at 30 days. To further characterize hs‐HP performance in these subgroups, we also evaluated rates of 30‐day major adverse cardiovascular events (MACE: all‐cause death, MI, and coronary revascularization), OCT, and early discharge (discharge from the ED without OCT).

## 2 Methods

### 2.1 Study Design

We conducted a subgroup analysis of the hs‐HP implementation study, a pre−post interrupted time series conducted at five US EDs which prospectively accrued patients from January 2019 to February 2022. This study was approved by the institutional review board with a waiver of informed consent. Methods of the hs‐HP implementation study have been previously described [5]. The Strengthening the Reporting of Observational Studies in Epidemiology (STROBE) guidelines helped direct the research and reporting processes [18].

### 2.2 Study Setting and Population

This study took place at five hospitals in North Carolina: Wake Forest Baptist Medical Center (WFBMC), with approximately 114 000 ED visits per year; Davie Medical Center (DMC), with approximately 20 000 visits per year; Lexington Medical Center (LMC), with approximately 37 000 ED visits per year; High Point Medical Center (HPMC), with approximately 80 000 visits per year; and Wilkes Medical Center (WMC), with approximately 35 000 visits annually. The pre‐implementation cohort enrolled patients at each site from January 1, 2019, through April 30, 2020. As the included medical centers began implementation of the hs‐HP, a 6‐month wash‐in phase started on May 1, 2020. Patients were thereafter accrued into the post‐implementation cohort from November 1, 2020, through February 28, 2022. Patients were included based on the date of their initial ED visit, with later visits for chest pain considered recurrent care. If a patient was transferred within the network to another site, care at the receiving hospital was considered part of their index encounter.

The study accrued adult ( ≥ 18 years old) ED patients investigated for possible ACS who either had a chief complaint of chest pain and at least one troponin ordered, or another complaint concerning for ACS (e.g., abdominal pain with shortness of breath) where the provider completed a HEART pathway assessment in the electronic health record (EHR). Patients with evidence of ST‐segment elevation myocardial infarction (STEMI) were excluded.

### 2.3 Data Collection

For both the pre‐ and post‐implementation periods, index encounter data was extracted from each medical center's EHR (Clarity‐Epic Systems Corporation, Verona, WI). Patient demographics, including sex and race, were self‐reported and extracted from the EHR. Pre‐validated structured EHR variables or diagnoses and procedure codes (CPT, ICD9, and ICD10) were used to obtain patient demographics, comorbidities, cardiovascular risk factors, troponin results, dispositions, diagnoses, and vital status [19, 20, 21, 22, 23]. HEART pathway assessments were completed prospectively by the patient's treating provider using an application integrated into the EHR (Impathiq Inc., Raleigh, NC). The North Carolina State Center for Health Statistics death index data was used as well to supplement vital statistics [24].

### 2.4 High‐Sensitivity Troponin Pathway Implementation

During the pre‐implementation period, the HEART pathway (Supporting Information S1: eFigure 1) was used to risk stratify patients with acute chest pain at each site [2, 19]. The HEART pathway included serial troponin measurements at 0‐ and 3‐h with the ADVIA Centaur platform TnI‐Ultra assay (Siemens, Munich, Germany) or the Access AccuTnI+3 assay (Beckman Coulter, Brea, CA). Patients with HEAR scores ≤ 3 and without elevated troponin measures were classified as low‐risk and recommended for discharge from the ED without OCT. Patients were classified as non‐low risk and recommended for further testing or admission if they presented with any of the following: a HEAR score ≥ 4, ischemic changes on ECG, an elevated troponin, or known CAD. Consistent with prior applications of the HEART pathway, known CAD is defined as any prior MI, coronary revascularization, or obstructive coronary stenosis ≥ 70% [2, 19].

After the initial pre‐implementation period concluded, the hs‐HP was integrated into each medical center's EHR in place of the original HEART pathway as an interactive ADP. Both the contemporary HEART pathway and the hs‐HP gave providers a pop‐up alert for all adult patients with chest pain and at least one troponin ordered. Both pathways were also integrated into the study‐specific EHR flowsheet, allowing providers to manually access the original HEART pathway or hs‐HP in patients presenting with symptoms concerning for ACS other than chest pain (e.g., shortness of breath, diaphoresis with left arm pain, etc.).

During the post‐implementation period, the hs‐HP (Supporting Information S1: eFigure 2) was used as the standard risk stratification strategy at each site. There was a 6‐month wash‐in period to allow for training and providers to become accustomed to the new hs‐HP. The hs‐HP has several key differences from the original HEART pathway. First, serial hs‐cTnI at 0‐ and 2‐h were incorporated with the Access 2 assay (Beckman Coulter, Brea, CA), which has a 99th percentile upper reference limit (URL) of 18 ng/L and 10% coefficient of variation at 4 ng/L. Second, a “one‐and‐done” approach was used to rule‐out patients with a nonischemic ECG, no prior CAD history, a HEAR score ≤ 3, and chest pain onset > 3 h with a single troponin measure < 4 ng/L. Patients with a HEAR score ≤ 3 who had chest pain for < 3 h or an initial troponin > 4 ng/L were assigned to rule‐in, rule‐out, and observation groups based on absolute hs‐cTnI cut points and a delta value from serial measures. Because this study was implemented in real‐world EDs, not all providers strictly adhered to the hs‐HP protocol when identifying patients for early discharge. Therefore, any patients with a HEAR score < 4 who were discharged from the ED with a single non‐elevated hs‐cTnI but did not otherwise meet the “one‐and‐done” criteria were defined as nonadherent rule‐outs. Finally, patients with a HEAR score from 4 to 6 but without ischemic ECG findings or hs‐cTnI above the URL were eligible for early discharge from the ED with rapid (within 72 h) outpatient follow‐up with cardiology through the “outpatient pathway.”

### 2.5 Outcomes

The primary safety outcome was 30‐day all‐cause death or MI, including the index visit. The primary effectiveness outcome was 30‐day hospitalization, inclusive of the index encounter. We also evaluated index and 30‐day MACE (all‐cause death, MI, or coronary revascularization), as well as the individual MACE components. Revascularization included any percutaneous coronary intervention (PCI) with or without stenting and/or coronary artery bypass grafting (CABG). We defined hospitalization as either an inpatient admission, a transfer, or an admission to an observation unit. Additional secondary outcomes included OCT and early discharge rate. OCT included stress testing, CCTA, and invasive angiography. Early discharge was defined as being discharged from the ED without OCT.

### 2.6 Statistical Analysis

Demographics and risk factors were compared pre‐ versus post‐implementation overall and within each subgroup using chi‐square or Wilcoxon rank sum tests, as appropriate. Unadjusted logistic regression was used to evaluate the association between the implementation cohort and each outcome within each subgroup separately. Models were then adjusted for the following potential confounders that were selected a priori: age, sex (race subgroups only), race (sex subgroups only), ED location, ethnicity, insurance status, and cardiovascular risk factors (prior CAD, diabetes, hyperlipidemia, hypertension, and smoking). To test for significant differences in implementation between sex and race, logistic regression models were fit using the overall population including sex by implementation cohort or race by implementation cohort interaction terms. The same potential confounders were included in these models. Unadjusted and adjusted odds ratios (aOR) for the post‐ versus pre‐implementation cohorts were computed and reported with corresponding 95% confidence intervals (CIs) for each logistic model. Safety and effectiveness outcomes were also compared between the pre‐ and post‐implementation cohorts within each subgroup using two‐sample proportion tests. Absolute percentage differences along with 95% CIs were also calculated (Supporting Information S1: eTables 1a and 1b).

Post‐implementation, efficacy, sensitivity, specificity, positive predictive value (PPV), negative predictive value (NPV), positive likelihood ratio (LR+), and negative likelihood ratio (LR−) for the hs‐HP for 30‐day death or MI were calculated within each sex and race subgroup. Efficacy was defined as the proportion of patients identified for early discharge from the ED. For efficacy, sensitivity, specificity, NPV, and PPV, exact 95% CIs were computed. For LR+ and LR−, the method of Simel et al. was used for 95% CIs [25]. Specificity, LR+, and PPV were calculated by comparing the rule‐in group versus observation + rule‐out group. Efficacy, sensitivity, NPV, and LR− were calculated individually for each of the following rule‐out groups: the “one‐and‐done” rule‐out, the 0/2 h rule‐out, the outpatient pathway, and nonadherent rule‐out groups. The combination of “one‐and‐done” + 0/2 h rule‐out, the combination of ”one‐and‐done” + 0/2 h + nonadherent rule‐out, and the combination of all four of these groups were also considered. The proportion of safety events and effectiveness outcomes in those patients ruled‐out (“one‐and‐done” + 0/2 h + nonadherent rule‐out) in the post‐implementation cohort were compared between sexes and races using Fisher's exact or chi‐square tests as appropriate.

In addition, individual components of the hs‐HP were compared between subgroups using chi‐square tests for those patients with a complete hs‐HP assessment in the post‐implementation period (Supporting Information S1: eTables 3a and 3b). Patients were considered to have a complete hs‐HP assessment provided there was sufficient data available to make a risk determination, so a complete assessment does not require having data on each component. A sensitivity analysis was also conducted to further evaluate key safety and effectiveness outcomes within subgroups between the pre‐ and post‐implementation cohorts. For this analysis, only those patients with complete HEART pathway assessments (either the original HEART pathway assessment in the pre‐implementation cohort or the hs‐HP assessment in the post‐implementation cohort) were included (Supporting Information S1: eTables 4a and 4b).

## 3 Results

### 3.1 Patients

This analysis accrued 26 126 total patients. The population was 52.7% (13 767/26 126) women and 64.7% (16 908/26 126) White, with a median age of 54 years (IQR: 42−66). In the pre‐implementation cohort, there were 12 317 patients, and 13 809 patients in the post‐implementation cohort. The pre‐implementation cohort was 52.4% (6460/12 317) women and 60.9% (7497/12 317) White, while the post‐implementation cohort was 52.9% (7307/13 809) women and 64.9% (8961/13 809) White. Characteristics of the pre‐ and post‐implementation cohorts are summarized by sex and race in Table 1. The 30‐day death or MI rate for the overall cohort inclusive of index was 7.2% (1893/26 126) and the rate of 30‐day MACE was 8.0% (2092/26 126).

**Table 1:** Characteristics of patients in the pre‐ and post‐implementation cohorts by sex and race.

Patient characteristics	Pre‐implementation (*N* = 12 317)			Post‐implementation (*N* = 13 809)			Total (*N* = 26 126)		
	Female (*N* = 6460) (%)	Male (*N* = 5857) (%)	*p* value	Female (*N* = 7307) (%)	Male (*N* = 6502) (%)	*p* value	Female (*N* = 13 767) (%)	Male (*N* = 12 359) (%)	*p* value
Age, years (median‐IQR)	54 (67−43)	55 (66−44)	0.78	54 (67−41)	54 (66−42)	0.33	54 (67−42)	55 (66−43)	0.36
Race			< 0.001			< 0.001			< 0.001
White	4018 (62.2)	3929 (67.1)		4636 (63.5)	4325 (66.5)		8654 (62.9)	8254 (66.8)	
Non‐White	2442 (37.8)	1928 (32.9)		2671 (36.6)	2177 (33.5)		5113 (37.1)	4105 (33.2)	
Ethnicity			0.02			0.67			0.05
Hispanic/Latino	420 (6.5)	320 (5.5)		493 (6.8)	427 (6.6)		913 (6.6)	747 (6.0)	
Site			0.57			0.03			0.05
WFBMC	2704 (41.9)	2411 (41.2)		2544 (34.8)	2354 (36.2)		5248 (38.1)	4765 (38.6)	
DMC	695 (10.8)	594 (10.1)		872 (11.9)	677 (10.4)		1567 (11.4)	1271 (10.3)	
HPMC	1789 (27.7)	1688 (28.8)		2063 (28.2)	1844 (28.4)		3852 (28.0)	3532 (28.6)	
WMC	331 (5.1)	305 (5.2)		889 (12.2)	831 (12.8)		1220 (8.9)	1136 (9.2)	
LMC	941 (14.6)	859 (14.7)		939 (12.9)	796 (12.2)		1880 (13.7)	1655 (13.4)	
Insurance status			< 0.001			< 0.001			< 0.001
Medicaid	1011 (15.7)	784 (13.4)		1026 (14.0)	751 (11.6)		2037 (14.8)	1535 (12.4)	
Medicare	2448 (37.9)	1944 (33.2)		2493 (34.1)	1948 (30.0)		4941 (35.9)	3892 (31.5)	
Private	2000 (31.0)	1956 (33.4)		3004 (41.1)	2690 (41.4)		5004 (36.4)	4646 (37.6)	
Uninsured/self‐pay	1001 (15.5)	1173 (20.0)		784 (10.7)	1113 (17.1)		1785 (13.0)	2286 (18.5)	
Risk factors									
Prior CAD	1086 (16.8)	1767 (30.2)	< 0.001	1064 (14.6)	1600 (24.6)	< 0.001	2150 (15.6)	3367 (27.2)	< 0.001
Diabetes	1802 (27.9)	1643 (28.1)	0.85	1861 (25.5)	1661 (25.6)	0.92	3663 (26.6)	3304 (26.7)	0.82
Hyperlipidemia	2706 (41.9)	2677 (45.7)	< 0.001	2853 (39.0)	2711 (41.7)	0.002	5559 (40.4)	5388 (43.6)	< 0.001
Hypertension	3969 (61.4)	3748 (64.0)	0.003	4093 (56.0)	3831 (58.9)	< 0.001	8062 (58.6)	7579 (61.3)	< 0.001
Smoking	1618 (25.1)	1951 (33.3)	< 0.001	1514 (20.7)	1795 (27.6)	< 0.001	3132 (22.8)	3746 (30.3)	< 0.001
Obesity (BMI ≥ 30)	2356 (53.3)	1822 (43.2)	< 0.001	2188 (53.0)	1663 (43.5)	< 0.001	4544 (53.2)	3485 (43.3)	< 0.001

	**Non‐White (*N*** = **4370) (%)**	**White (*N*** = **7947) (%)**		**Non‐White (*N*** = **4848) (%)**	**White (*N*** = **8961) (%)**		**Non‐White (*N*** = **9218) (%)**	**White (*N*** = **16 908) (%)**	
Age, years (median‐IQR)	51 (62−40)	57 (70−46)	< 0.001	50 (61−38)	56 (69−43)	< 0.001	51 (61−39)	57 (69−45)	< 0.001
Sex			< 0.001			< 0.001			< 0.001
Female	2442 (55.9)	4018 (50.6)		2671 (55.1)	4636 (51.7)		5113 (55.5)	8654 (51.2)	
Male	1928 (44.1)	3929 (49.4)		2177 (44.9)	4325 (48.3)		4105 (44.5)	8254 (48.8)	
Ethnicity			< 0.001			< 0.001			< 0.001
Hispanic/Latino	672 (15.4)	68 (0.86)		837 (17.3)	83 (0.93)		1509 (16.4)	151 (0.89)	
Site			< 0.001			< 0.001			< 0.001
WFBMC	2373 (54.3)	2742 (34.5)		2302 (47.5)	2596 (29.0)		4675 (50.7)	5338 (31.6)	
DMC	237 (5.4)	1052 (13.2)		339 (7.0)	1210 (13.5)		576 (6.3)	2262 (13.4)	
HPMC	1297 (29.7)	2180 (27.4)		1569 (32.4)	2338 (26.1)		2866 (31.1)	4518 (26.7)	
WMC	76 (1.7)	560 (7.1)		197 (4.1)	1523 (17.0)		273 (3.0)	2083 (12.3)	
LMC	387 (8.9)	1413 (17.8)		441 (9.1)	1294 (14.4)		828 (9.0)	2707 (16.0)	
Insurance status			< 0.001			< 0.001			< 0.001
Medicaid	796 (18.2)	999 (12.6)		759 (15.7)	1018 (11.4)		1555 (16.9)	2017 (11.9)	
Medicare	1232 (28.2)	3160 (39.8)		1235 (25.5)	3206 (35.8)		2467 (26.8)	6366 (37.7)	
Private	1240 (28.4)	2716 (34.2)		1924 (39.7)	3770 (42.1)		3164 (34.3)	6486 (38.4)	
Uninsured/self‐pay	1102 (25.2)	1072 (13.5)		930 (19.2)	967 (10.8)		2032 (22.0)	2039 (12.1)	
Risk factors									
Prior CAD	705 (16.1)	2148 (27.0)	< 0.001	607 (12.5)	2057 (23.0)	< 0.001	1312 (14.2)	4205 (24.9)	< 0.001
Diabetes	1347 (30.8)	2098 (26.4)	< 0.001	1369 (28.2)	2153 (24.0)	< 0.001	2716 (29.5)	4251 (25.1)	< 0.001
Hyperlipidemia	1585 (36.3)	3798 (47.8)	< 0.001	1625 (33.5)	3939 (44.0)	< 0.001	3210 (34.8)	7737 (45.8)	< 0.001
Hypertension	2779 (63.6)	4938 (62.1)	0.11	2779 (57.3)	5145 (57.4)	0.92	5558 (60.3)	10 083 (59.6)	0.30
Smoking	1200 (27.5)	2369 (29.8)	0.01	1100 (22.7)	2209 (24.7)	0.01	2300 (25.0)	4578 (27.1)	< 0.001
Obesity (BMI ≥ 30)	1589 (51.3)	2589 (46.8)	< 0.001	1458 (52.8)	2393 (46.1)	< 0.001	3047 (52.0)	4982 (46.4)	< 0.001

Abbreviations: BMI, body mass index; CAD, coronary artery disease; DMC, Davie Medical Center; HPMC, High Point Medical Center; IQR, interquartile range; LMC, Lexington Medical Center; WFMBC, Wake Forest Baptist Medical Center; WMC, Wilkes Medical Center.

### 3.2 Safety

At 30 days, death or MI occurred in 4.6% (335/7307) of women in the post‐implementation cohort compared to 5.4% (347/6460) pre‐implementation (aOR: 0.92 [95% CI: 0.78−1.09]). In men, 30‐day death or MI occurred in 9.4% (610/6502) post‐implementation versus 10.3% (601/5857) pre‐implementation (aOR: 1.04 [95% CI: 0.91−1.18]). The interaction between implementation cohort and sex was not significant for 30‐day death or MI (*p* = 0.24).

**Figure 1:**
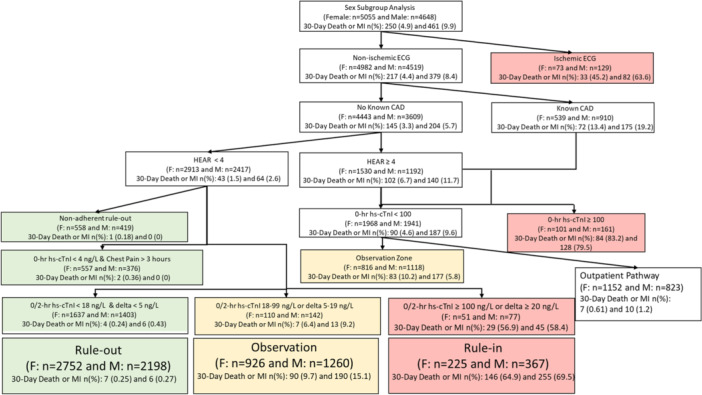
Sex subgroup analysis for patients with a complete hs‐HP assessment. CAD, coronary artery disease; ECG, electrocardiogram; HEAR, history, ECG, age, and risk; hs‐cTnI, high‐sensitivity cardiac troponin I; MI, myocardial infarction.

**Figure 2:**
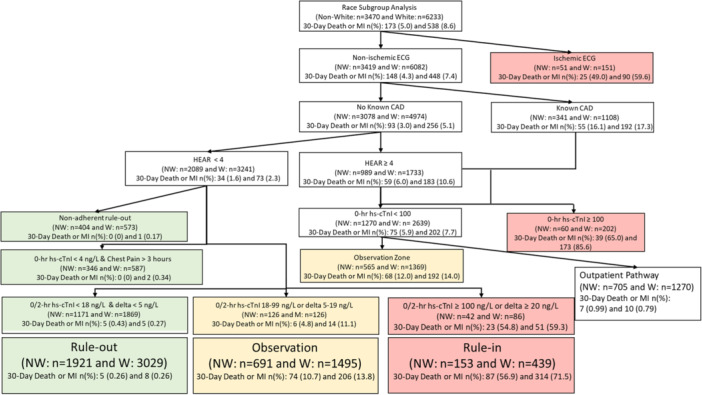
Race subgroup analysis for patients with a complete hs‐HP assessment. CAD, coronary artery disease; ECG, electrocardiogram; HEAR, history, ECG, age, and risk; hs‐cTnI, high‐sensitivity cardiac troponin I; MI, myocardial infarction.

Among White patients, 30‐day death or MI occurred in 8.2% (731/8961) of patients post‐implementation versus 8.9% (704/7947) of patients pre‐implementation (aOR: 1.03 [95% CI: 0.92−1.16]). In non‐White patients, 30‐day death or MI occurred in 4.4% (214/4848) of patients versus 5.6% (244/4370) of patients in post‐ and pre‐implementation cohorts, respectively (aOR: 0.91 [95% CI: 0.74−1.11]). The interaction between the implementation cohort and race was not significant for 30‐day death or MI (*p *= 0.22). Study outcomes by sex and race are summarized in Table 2 and Figures 1 and 2.

**Table 2:** Proportion of female versus male and White versus non‐White patients with events in the pre‐ and post‐implementation cohorts.

Outcomes	Female			Male			Interaction
	Pre (*N* = 6460) (%)	Post (*N* = 7307) (%)	Adjusted odds ratio (95% CI)^a^	Pre (*N* = 5857) (%)	Post (*N* = 6502) (%)	Adjusted odds ratio (95% CI)^a^	Sex × implementation cohort *p* value
*Safety*							
Index							
Death^b^	13 (0.20)	15 (0.21)	1.07 (0.51−2.26)	20 (0.34)	31 (0.48)	1.42 (0.80−2.49)	0.52
MI	291 (4.5)	280 (3.8)	0.92 (0.77−1.10)	529 (9.0)	523 (8.0)	1.02 (0.89−1.16)	0.38
Revascularization	128 (2.0)	127 (1.7)	1.00 (0.77−1.30)	316 (5.4)	273 (4.2)	0.91 (0.76−1.09)	0.61
Death or MI	300 (4.6)	289 (4.0)	0.92 (0.77−1.10)	540 (9.2)	545 (8.4)	1.04 (0.91−1.19)	0.28
MACE	321 (5.0)	308 (4.2)	0.92 (0.78−1.09)	597 (10.2)	586 (9.0)	1.01 (0.88−1.15)	0.38
30‐day follow‐up							
Death^c^	34 (0.53)	35 (0.48)	0.99 (0.61−1.59)	48 (0.82)	48 (0.74)	0.94 (0.63−1.41)	0.91
MI^c^	39 (0.60)	39 (0.53)	1.00 (0.64−1.57)	64 (1.1)	63 (0.97)	1.01 (0.71−1.44)	0.90
Revascularization^d^	29 (0.45)	24 (0.33)	0.82 (0.47−1.42)	70 (1.2)	55 (0.85)	0.80 (0.56−1.15)	0.96
Death or MI	73 (1.1)	70 (0.96)	0.92 (0.66−1.29)	108 (1.8)	105 (1.6)	0.94 (0.71−1.24)	0.81
MACE	82 (1.3)	82 (1.1)	0.96 (0.70−1.32)	143 (2.4)	133 (2.1)	0.91 (0.71−1.17)	0.87
30‐day (index + follow‐up)							
Death	47 (0.73)	50 (0.68)	1.00 (0.66−1.50)	68 (1.2)	79 (1.2)	1.07 (0.77−1.50)	0.69
MI	313 (4.9)	300 (4.1)	0.92 (0.77−1.09)	554 (9.5)	552 (8.5)	1.03 (0.90−1.17)	0.31
Revascularization	156 (2.4)	149 (2.0)	0.95 (0.75−1.21)	380 (6.5)	322 (5.0)	0.89 (0.75−1.04)	0.65
Death or MI	347 (5.4)	335 (4.6)	0.92 (0.78−1.09)	601 (10.3)	610 (9.4)	1.04 (0.91−1.18)	0.24
MACE	376 (5.8)	363 (5.0)	0.93 (0.79−1.08)	688 (11.8)	665 (10.2)	0.98 (0.87−1.11)	0.51
*Effectiveness*							
Index							
Early discharge	3040 (47.1)	4924 (67.4)	**2.31 (2.14−2.50)**	2343 (40.0)	3856 (59.3)	**2.14 (1.97−2.32)**	0.20
Hospitalization	2713 (42.0)	1874 (25.7)	**0.47 (0.43−0.51)**	2922 (49.9)	2164 (33.3)	**0.50 (0.46−0.55)**	0.24
Objective cardiac testing	1296 (20.1)	689 (9.4)	**0.43 (0.39−0.48)**	1396 (23.8)	924 (14.2)	**0.56 (0.50−0.61)**	**< 0.001**
30‐day follow‐up							
Hospitalization	335 (5.2)	373 (5.1)	1.07 (0.91−1.25)	452 (7.7)	433 (6.7)	0.93 (0.81−1.07)	0.23
Objective cardiac testing	267 (4.1)	280 (3.8)	0.88 (0.74−1.05)	275 (4.7)	302 (4.6)	0.98 (0.83−1.16)	0.39
30‐day (index + follow‐up)							
Hospitalization	2810 (43.5)	2035 (27.9)	**0.50 (0.46−0.54)**	3043 (52.0)	2304 (35.4)	**0.51 (0.47−0.56)**	0.69
Objective cardiac testing	1506 (23.3)	928 (12.7)	**0.49 (0.45−0.54)**	1599 (27.3)	1163 (17.9)	**0.61 (0.55−0.67)**	**< 0.001**

Outcomes	White			Non‐White			Interaction
	**Pre (*N*** = **7947) (%)**	**Post (*N*** = **8961) (%)**	**Adjusted odds ratio (95% CI)** a	**Pre (*N*** = **4370) (%)**	**Post (*N*** = **4848) (%)**	**Adjusted odds ratio (95% CI)** a	**Race** × **implementation cohort *p* value**
*Safety*							
Index							
Death^e^	23 (0.29)	38 (0.42)	1.54 (0.92−2.59)	10 (0.23)	8 (0.17)	0.73 (0.29−1.85)	0.17
MI	605 (7.6)	627 (7.0)	1.03 (0.91−1.17)	215 (4.9)	176 (3.6)	0.84 (0.68−1.05)	0.08
Revascularization	357 (4.5)	331 (3.7)	0.96 (0.81−1.13)	87 (2.0)	69 (1.4)	0.90 (0.64−1.27)	0.57
Death or MI	620 (7.8)	652 (7.3)	1.05 (0.93−1.19)	220 (5.0)	182 (3.8)	0.85 (0.69−1.05)	0.07
MACE	682 (8.6)	701 (7.8)	1.03 (0.92−1.16)	236 (5.4)	193 (4.0)	0.83 (0.68−1.03)	0.07
30‐day follow‐up							
Death^f^	66 (0.83)	64 (0.71)	0.92 (0.65−1.30)	16 (0.37)	19 (0.39)	1.09 (0.55−2.13)	0.64
MI^f^	81 (1.0)	79 (0.88)	0.97 (0.71−1.33)	22 (0.50)	23 (0.47)	1.11 (0.61−2.01)	0.72
Revascularization^f^	82 (1.0)	63 (0.70)	0.78 (0.56−1.08)	17 (0.39)	16 (0.33)	0.98 (0.49−1.96)	0.49
Death or MI^g^	143 (1.8)	134 (1.5)	0.92 (0.73−1.17)	38 (0.87)	41 (0.85)	1.10 (0.70−1.73)	0.49
MACE	180 (2.3)	167 (1.9)	0.89 (0.72−1.11)	45 (1.0)	48 (0.99)	1.10 (0.72−1.68)	0.36
30‐day (index + follow‐up)							
Death^h^	89 (1.1)	102 (1.1)	1.08 (0.81−1.44)	26 (0.59)	27 (0.56)	0.97 (0.56−1.67)	0.67
MI	641 (8.1)	659 (7.4)	1.02 (0.91−1.15)	226 (5.2)	193 (4.0)	0.89 (0.72−1.10)	0.20
Revascularization	435 (5.5)	387 (4.3)	0.91 (0.78−1.06)	101 (2.3)	84 (1.7)	0.92 (0.67−1.25)	> 0.99
Death or MI	704 (8.9)	731 (8.2)	1.03 (0.92−1.16)	244 (5.6)	214 (4.4)	0.91 (0.74−1.11)	0.22
MACE	798 (10.0)	799 (8.9)	0.99 (0.88−1.10)	266 (6.1)	229 (4.7)	0.88 (0.72−1.07)	0.29
*Effectiveness*							
Index							
Early discharge	3301 (41.5)	5590 (62.4)	**2.30 (2.15−2.47)**	2082 (47.6)	3190 (65.8)	**2.10 (1.91−2.31)**	0.07
Hospitalization	3857 (48.5)	2778 (31.0)	**0.47 (0.44−0.51)**	1778 (40.7)	1260 (26.0)	**0.50 (0.45−0.55)**	0.23
Objective cardiac testing	1842 (23.2)	1154 (12.9)	**0.52 (0.47−0.56)**	850 (19.5)	459 (9.5)	**0.44 (0.39−0.50)**	0.05
30‐day follow‐up							
Hospitalization	550 (6.9)	566 (6.3)	0.98 (0.86−1.11)	237 (5.4)	240 (5.0)	1.04 (0.86−1.25)	0.71
Objective cardiac testing	417 (5.2)	430 (4.8)	0.90 (0.78−1.03)	125 (2.9)	152 (3.1)	1.04 (0.82−1.34)	0.26
30‐day (index + follow‐up)							
Hospitalization	4000 (50.3)	2966 (33.1)	**0.49 (0.45−0.52)**	1853 (42.4)	1373 (28.3)	**0.53 (0.48−0.59)**	0.10
Objective cardiac testing	2155 (27.1)	1498 (16.7)	**0.57 (0.52−0.61)**	950 (21.7)	593 (12.2)	**0.50 (0.45−0.57)**	0.11

*Note:* Bold values are statistically significant.

Abbreviations: 95% CI, 95% confidence interval; MACE, major adverse cardiac event; MI, myocardial infarction.

^a^ Models were adjusted for age, sex or race, ethnicity, ED location, insurance status, smoking, prior CAD, hypertension, hyperlipidemia, and diabetes unless otherwise stated.

^b^ Model was adjusted for age, race, and prior CAD.

^c^ Models were adjusted for age, race, prior CAD, hypertension, diabetes, hyperlipidemia, and smoking.

^d^ Models were adjusted for age, race, prior CAD, hypertension, diabetes, and hyperlipidemia.

^e^ Models were adjusted for age and sex.

^f^ Models were adjusted for age, sex, prior CAD, and hypertension.

^g^ Models were adjusted for age, sex, prior CAD, hypertension, hyperlipidemia, diabetes, smoking, and insurance status.

^h^ Models were adjusted for age, sex, prior CAD, hypertension, hyperlipidemia, and diabetes.

In the post‐implementation cohort, 70.3% (9703/13 809) of patients had complete hs‐HP assessments. Of those with complete assessments, 56.7% (5055/9703) were female and 64.2% (6233/9703) were White. Among these patients, 7.1% more women were classified to the rule‐out group (“one‐and‐done” + 0/2 h + nonadherent rule‐out) than men (54.4% [2752/5055] vs. 47.3% [2198/4648], *p *< 0.001). The outpatient pathway criteria also identified more additional women for early discharge than men (22.8% [1152/5055] vs. 17.7% [823/4648], *p *< 0.001). Among patients classified to the combined rule‐out and outpatient pathway group, 0.4% (14/3904) of women and 0.5% (16/3021) of men had 30‐day death or MI (*p *= 0.36). Similarly, 48.6% (3029/6233) of White patients with complete assessments in the post‐implementation cohort were classified to the rule‐out group, 6.8% less than non‐White patients (55.4% [1921/3470], *p *< 0.001). The outpatient pathway identified an additional 20.4% (1270/6233) of White patients and 20.3% (705/3470) of non‐White patients for early discharge (*p *= 0.96). Among those classified to the rule‐out and outpatient pathway group, 0.4% (18/4299) of White patients and 0.5% (12/2626) of non‐White patients had 30‐day death or MI (*p *= 0.85). Test characteristics analyzing the performance of the hs‐HP stratified by sex and race are summarized in Table 3. Patient outcomes among the rule‐out group are summarized in Supporting Information S1: eTable 2. Figures 1 and 2 graphically portray how patients were categorized by the hs‐HP and their outcomes. Score components of the hs‐HP assessment are described in Supporting Information S1: eTables 3a and 3b.

**Table 3:** Test characteristics of the high‐sensitivity HEART pathway for detection of death or MI at 30 days among the post‐implementation cohort based on sex and race.

	Sensitivity (95% CI)	NPV (95% CI)	LR− (95% CI)	Efficacy^a^ (95% CI)
*Male*				
Rule‐out: one‐and‐done	100 (99.2−100)	100 (99.0−100)	0 (0−NA)	8.1 (7.3−8.9)
Rule‐out: 0/2 h rule	98.7 (97.2−99.5)	99.6 (99.1−99.8)	0.04 (0.02−0.09)	30.2 (28.9−31.5)
Nonadherent rule‐out	100 (99.2−100)	100 (99.1−100)	0 (0−NA)	9.0 (8.2−9.9)
Rule‐out (one‐and‐done + 0/2)	98.7 (97.2−99.5)	99.7 (99.3−99.9)	0.03 (0.01−0.07)	38.3 (36.9−39.7)
Rule‐out (one‐and‐done + 0/2 + nonadherent)	98.7 (97.2−99.5)	99.7 (99.4−99.9)	0.03 (0.01−0.06)	47.3 (45.8−48.7)
Outpatient pathway	97.8 (96.0−99.0)	98.8 (97.8−99.4)	0.11 (0.06−0.21)	17.7 (16.6−18.8)
Outpatient pathway + rule‐out (one‐and‐done + 0/2 + nonadherent)	96.5 (94.4−98.0)	99.5 (99.1−99.7)	0.05 (0.03−0.08)	65.0 (64.6−66.4)
	**Specificity (95% CI)**	**PPV (95% CI)**	**LR+ (95% CI)**	**Rule‐in proportion (95% CI)**
Rule‐in	97.3 (96.8−97.8)	69.5 (64.5−74.2)	20.7 (16.9−25.3)	7.9 (7.1−8.7)
*Female*	**Sensitivity (95% CI)**	**NPV (95% CI)**	**LR− (95% CI)**	**Efficacy (95% CI)**
Rule‐out: one‐and‐done	99.2 (97.1−99.9)	99.6 (98.7−100)	0.07 (0.02−0.28)	11.0 (10.2−11.9)
Rule‐out: 0/2 h rule	98.4 (96.0−99.6)	99.8 (99.4−99.9)	0.05 (0.02−0.13)	32.4 (31.1−33.7)
Nonadherent rule‐out	99.6 (97.8−100)	99.8 (99.0−100)	0.04 (0.01−0.24)	11.0 (10.2−11.9)
Rule‐out (one‐and‐done + 0/2)	97.6 (94.8−99.1)	99.7 (99.4−99.9)	0.05 (0.02−0.12)	43.4 (42.0−44.8)
Rule‐out (one‐and‐done + 0/2 + nonadherent	97.2 (94.3−98.9)	99.7 (99.5−99.9)	0.05 (0.02−0.10)	54.4 (53.1−55.8)
Outpatient pathway	97.2 (94.3−98.9)	99.4 (98.8−99.8)	0.12 (0.06−0.24)	22.8 (21.6−24.0)
Outpatient pathway + rule‐out (one‐and‐done + 0/2 + nonadherent)	94.4 (90.8−96.9)	99.6 (99.4−99.8)	0.07 (0.04−0.12)	77.2 (76.0−78.4)
	**Specificity (95% CI)**	**PPV (95% CI)**	**LR+ (95% CI)**	**Rule‐in proportion**
Rule‐in	98.4 (98.0−98.7)	64.9 (58.3−71.1)	35.5 (27.9−45.3)	4.5 (3.9−5.1)
*White*	**Sensitivity (95% CI)**	**NPV (95% CI)**	**LR− (95% CI)**	**Efficacy (95% CI)**
Rule‐out: one‐and‐done	99.6 (98.7−100)	99.7 (98.8−100)	0.04 (0.01−0.15)	9.4 (8.7−10.2)
Rule‐out: 0/2 h rule	99.1 (97.8−99.7)	99.7 (99.4−99.9)	0.03 (0.01−0.07)	30.0 (28.8−31.1)
Nonadherent rule‐out	99.8 (99.0−100)	99.8 (99.0−100)	0.02 (0.00−0.13)	9.2 (8.5−9.9)
Rule‐out (one‐and‐done + 0/2)	98.7 (97.3−99.5)	99.7 (99.4−99.9)	0.03 (0.01−0.06)	39.4 (38.2−40.6)
Rule‐out (one‐and‐done + 0/2 + nonadherent)	98.5 (97.1−99.4)	99.7 (99.5−99.9)	0.03 (0.01−0.06)	48.6 (47.3−49.8)
Outpatient pathway	98.1 (96.6−99.1)	99.2 (98.6−99.6)	0.08 (0.05−0.16)	20.4 (19.4−21.4)
Outpatient pathway + rule‐out (one‐and‐done + 0/2 + nonadherent	96.7 (94.8−98.0)	99.6 (99.3−99.8)	0.05 (0.03−0.07)	69.0 (67.8−70.1)
	**Specificity (95% CI)**	**PPV (95% CI)**	**LR+ (95% CI)**	**Rule‐in proportion (95% CI)**
Rule‐in	97.8 (97.4−98.2)	71.5 (67.1−75.7)	26.6 (22.0−32.1)	7.0 (6.4−7.7)
*Non‐White*	**Sensitivity (95% CI)**	**NPV (95% CI)**	**LR− (95% CI)**	**Efficacy (95% CI)**
Rule‐out: one‐and‐done	100 (97.9−100)	100 (98.9−100)	0 (0−NA)	10.0 (9.0−11.0)
Rule‐out: 0/2 h rule	97.1 (93.4−99.1)	99.6 (99.0−99.9)	0.08 (0.03−0.19)	33.7 (32.2−35.3)
Nonadherent rule‐out	100 (97.9−100)	100 (99.1−100)	0 (0−NA)	11.6 (10.6−12.8)
Rule‐out (one‐and‐done + 0/2)	97.1 (93.4−99.1)	99.7 (99.2−99.9)	0.06 (0.03−0.15)	43.7 (42.1−45.4)
Rule‐out (one‐and‐done + 0/2 + nonadherent)	97.1 (93.4−99.1)	99.7 (99.4−99.9)	0.05 (0.02−0.12)	55.4 (53.7−57.0)
Outpatient pathway	96.0 (91.8−98.4)	99.0 (98.0−99.6)	0.19 (0.09−0.40)	20.3 (19.0−21.7)
Outpatient pathway + rule‐out (one‐and‐done + 0/2 + nonadherent)	93.1 (88.2−96.4)	99.5 (99.2−99.8)	0.09 (0.05−0.15)	75.6 (74.2−77.1)
	**Specificity (95% CI)**	**PPV (95% CI)**	**LR+ (95% CI)**	**Rule‐in proportion (95% CI)**
Rule‐in	98.0 (97.5−98.4)	56.9 (48.6−64.8)	25.1 (19.0−33.3)	4.4 (3.8−5.1)

*Note:* Bold values are statistically significant.

Abbreviations: 95% CI, 95% confidence interval; LR−, negative likelihood ratio; LR+, positive likelihood ratio; NPV, negative predictive value; PPV, positive predictive value.

^a^ Efficacy is defined as the proportion of patients classified to the rule‐out group.

### 3.3 Effectiveness

Among women, the 30‐day hospitalization rate was 27.9% (2035/7307) post‐implementation, compared to 43.5% (2810/6460) pre‐implementation, a reduction of 15.6% (aOR: 0.50 [95% CI: 0.46−0.54]). Among men, the 30‐day hospitalization rate was 35.4% (2304/6502) post‐implementation versus 52.0% (3043/5857) pre‐implementation, a reduction of 16.6% (aOR: 0.51 [95% CI: 0.47−0.56]). The interaction between implementation cohort and sex was not significant for 30‐day hospitalization (*p* = 0.69).

Among White patients, the 30‐day hospitalization rate was 33.1% (2966/8961) in the post‐implementation cohort, compared to 50.3% (4000/7947) pre‐implementation. This was a reduction of 17.2% (aOR: 0.49 [95% CI: 0.45−0.52]). In non‐White patients, the 30‐day hospitalization rate was 28.3% (1373/4848) post‐implementation versus 42.4% (1853/4370) pre‐implementation. This was a reduction of 14.1% (aOR: 0.53 [95% CI: 0.48−0.59]). The interaction between implementation cohort and race regarding 30‐day hospitalization was not significant (*p *= 0.10). Effectiveness outcomes among all patients are summarized in Table 2 and Supporting Information S1: eTables 1a and 1b.

### 3.4 Secondary Endpoints

For women, early discharge occurred in 67.4% (4924/7307) post‐implementation versus 47.1% (3040/6460) pre‐implementation, a 20.3% increase (aOR: 2.31 [95% CI: 2.14−2.50]). Among men, early discharge was achieved in 59.3% (3856/6502) post‐implementation compared to 40.0% (2343/5857) pre‐implementation, an increase of 19.3% (aOR: 2.14 [95% CI: 1.97−2.32]. The interaction between the implementation cohort and sex was not significant (*p *= 0.20). For White patients, early discharge occurred in 62.4% (5590/8961) of post‐implementation patients versus 41.5% (3301/7947) of pre‐implementation patients, an increase of 20.9% (aOR: 2.30 [95% CI: 2.15−2.47]). For non‐White patients, early discharge occurred in 65.8% (3190/4848) of patients post‐implementation compared to 47.6% (2082/4370) of patients pre‐implementation, an increase of 18.2% (aOR: 2.10 [95% CI: 1.91−2.31]). The interaction between the implementation cohort and race was not significant (*p *= 0.07). Early discharge rates among all patients are summarized in Table 2 and among those with a complete hs‐HP assessment in Supporting Information S1: eTables 4a and 4b.

The rate of OCT at 30 days decreased by 10.6% among women after hs‐HP implementation, occurring in 12.7% (928/7307) of post‐implementation patients versus 23.3% (1506/6460) pre‐implementation (aOR: 0.49 [95% CI: 0.45−0.54]). For men, the 30‐day OCT rate decreased by 9.4%−17.9% (1163/6502) post‐implementation from 27.3% (1599/5857) pre‐implementation (aOR: 0.61 [95% CI: 0.55−0.67]). The interaction between the implementation cohort and sex was significant (*p *< 0.001). For White patients, the 30‐day OCT rate decreased by 10.4%, with a post‐implementation rate of 16.7% (1498/8961) versus a pre‐implementation rate of 27.1% (2155/7947) (aOR: 0.57 [95% CI: 0.53−0.61]). Among non‐White patients, the 30‐day OCT rate decreased by 9.5%, with a post‐implementation rate of 12.2% (593/4848) versus 21.7% (950/4370) pre‐implementation (aOR: 0.50 [95% CI: 0.45−0.57]). The interaction between the implementation cohort and race was not significant (*p *= 0.11). OCT rates among all patients are summarized in Table 3 and among those with complete assessment in Supporting Information S1: eTables 4a and 4b.

## 4 Discussion

This multisite study is the first to evaluate the performance of the hs‐HP in sex and race subgroups. Our primary finding is that the hs‐HP ADP is safe and effective for risk‐stratifying ED patients presenting with possible ACS in each sex and race subgroup. We demonstrated that the hs‐HP has an excellent safety profile, achieving a very low 30‐day death or MI rate among early discharge patients, regardless of sex or race. Implementation of the hs‐HP was associated with decreased hospitalizations and OCT and increased early discharges among men, women, White, and non‐White patients. Furthermore, nonsignificant interaction terms in our models suggest that the effect of the hs‐HP implementation on the primary safety and effectiveness outcomes did not differ based on sex and race. These data support the use of the hs‐HP for ED risk stratification in these key subgroups of patients with possible ACS.

The hs‐HP achieved an excellent safety profile across all subgroups. Among early discharge patients, the hs‐HP had an NPV > 99.5% for death or MI in men, women, White, and non‐White patients. Thus, in each subgroup, NPV was well above the commonly used chest pain ADP safety threshold of 99% NPV for death or MI [1]. Given this excellent diagnostic performance, emergency clinicians should feel comfortable using the hs‐HP regardless of sex or race. In addition, the NPV of the hs‐HP is notable, because the European Society of Cardiology (ESC) 0/1 h algorithm, which is commonly used and endorsed by guidelines, has not consistently met this safety threshold in US cohorts [26, 27]. Even when combined with the HEART score, the hs‐cTnT ESC 0/1 h algorithm has not been able to achieve adequate safety for routine use in any sex or race subgroup among US patients [28]. The high NPV of the hs‐HP, regardless of subgroup, may be because of its use of clinical variables, such as the patient's history, ECG findings, and risk factors along with serial hs‐cTn measures (unlike the ESC 0/1 h algorithm, which relies solely on hs‐cTn measures). This theory is supported by previous studies showing that adding clinical variables to the ESC 0/1 h algorithm improves its safety [29].

Implementation of the hs‐HP was associated with decreased hospitalizations at both the index encounter and at 30 days in each subgroup compared to the original HEART pathway. The interaction terms for both sex and race were not significant, indicating that hs‐HP implementation was associated with similar reductions in hospitalization in both sex and race subgroups. These data suggest that health systems still using the original HEART pathway could adopt the hs‐HP to improve ED throughput, decrease ED boarding, and reduce unnecessary hospitalizations in all subgroups.

Early discharge rates increased in each subgroup, with hs‐HP implementation being associated with a nearly 20% increase in early discharges, regardless of sex or race. In both pre‐ and post‐hs‐HP implementation cohorts, more women and non‐White patients had early discharges compared to men and White patients. These findings mirror prior studies demonstrating that women and non‐White patients are more likely to be stratified into a low‐risk or rule‐out group [10, 11]. The hs‐HP implementation was also associated with decreased rates of OCT across both sexes, with a greater reduction in OCT rates among women compared to men. Importantly, these differences did not translate into increased 30‐day adverse outcomes by sex or race. The infrequency of 30‐day adverse outcomes among women and non‐White patients suggests that the use of the hs‐HP did not underestimate risk in these subgroups or result in under‐testing.

Risk stratification differences observed in this study between men versus women and Whites versus non‐Whites may be related to population‐level differences in troponin values [30, 31, 32]. Males are well known to have higher 99th percentile hs‐cTn URL than women [32]. However, the hs‐HP utilizes a universal cut‐off regardless of patient sex or race. It also relies on delta hs‐cTnI values to mitigate this potential sex‐based disparity. It is possible that the integration of sex‐specific cut points into the hs‐hp could decrease sex‐based differences in early discharge and OCT rates.

## 5 Limitations

This study has limitations. Secular trends and provider maturation effects may affect validity of results. However, event rates were consistent across the pre‐ and post‐implementation period. While sex was self‐reported in the EHR, our study did not explore gender, a socially constructed variable that may not be accurately reflected in categorical terms [33]. The sex category does not take into account the experiences of nonbinary and transgender adults that may affect their cardiovascular health outcomes or the well‐documented bias they may experience on evaluation in the ED [34, 35]. Similarly, a patient may present as a separate race category than what they self‐identify with. However, self‐reported race and sex remains a standardized approach to identifying demographic data. Ideally, we could examine race in more granular terms than “non‐White.” However, very few patients identified as American Indian or Alaska Native, Asian, Native Hawaiian, or Other Pacific Islander. Therefore, we present our findings as White and non‐White. Our data also come from a mixture of five urban and suburban centers in North Carolina, a population that may not be generalizable to all US healthcare systems. Finally, this study was not designed to explain why differences in risk classification by subgroup occurred.

## 6 Conclusion

The hs‐HP achieved an excellent safety and effectiveness profile across all race and sex subgroups. It was associated with reduced hospitalizations and OCT while achieving very low rates of missed death or MI. Furthermore, the increased number of early discharges in all sex and race subgroups suggests that the hs‐HP could improve operational efficiencies within some health systems. These findings indicate that using the hs‐HP may aid clinicians in providing safe and effective care to patients with chest pain regardless of sex or race.

## Author Contributions

**Campbell Veasey:** investigation, methodology, and writing–original draft. **Anna C. Snavely:** conceptualization, data curation, formal analysis, investigation, methodology, resources, supervision, and writing–review and editing. **Zechariah L. Kearns:** investigation, methodology, and writing–original draft. **Nicklaus P. Ashburn:** conceptualization, data curation, investigation, methodology, supervision, and writing–review and editing. **Tara Hashemian:** methodology, data curation, formal analysis, and writing–review and editing. **Simon A. Mahler:** conceptualization, data curation, formal analysis, funding acquisition, investigation, methodology, project administration, resources, supervision, and writing–review and editing.

## Conflicts of Interest

Dr. Nicklaus P. Ashburn receives funding from NHLBI (K23HL169929) and AHRQ (R01HS029017). Dr. Anna C. Snavely receives funding from Abbott Laboratories, HRSA (1H2ARH399760100), and AHRQ (R01HS029017 and R21HS029234). Dr. Simon A. Mahler receives funding/support from Roche Diagnostics, Abbott Laboratories, QuidelOrtho, Siemens, Grifols, Pathfast, Beckman Coulter, Genetesis, Cytovale, National Foundation of Emergency Medicine, Duke Endowment, Brainbox, BlueJay Diagnostics, HRSA (1H2ARH399760100), and AHRQ (R01HS029017 and R21HS029234). He is a consultant for Roche, QuidelOrtho, Abbott, Siemens, Inflammatix, and Radiometer and is the Chief Medical Officer for Impathiq Inc. The other authors declare no conflicts of interest.

## Supporting information

Supporting information.

## Data Availability

Research data are not shared.
