# Supplementary material for: The High‐Sensitivity HEART Pathway Safely Reduces Hospitalizations Regardless of Sex or Race in a Multisite Prospective US Cohort
Source: Clin Cardiol. 2024 Oct 17;47(10):e70027. doi: 10.1002/clc.70027 (PMC11483562; doi:10.1002/clc.70027)
Supplement: Supplementary file 1 — Supporting information. [file CLC-47-e70027-s001.docx]

**SUPPLEMENTAL APPENDICES**
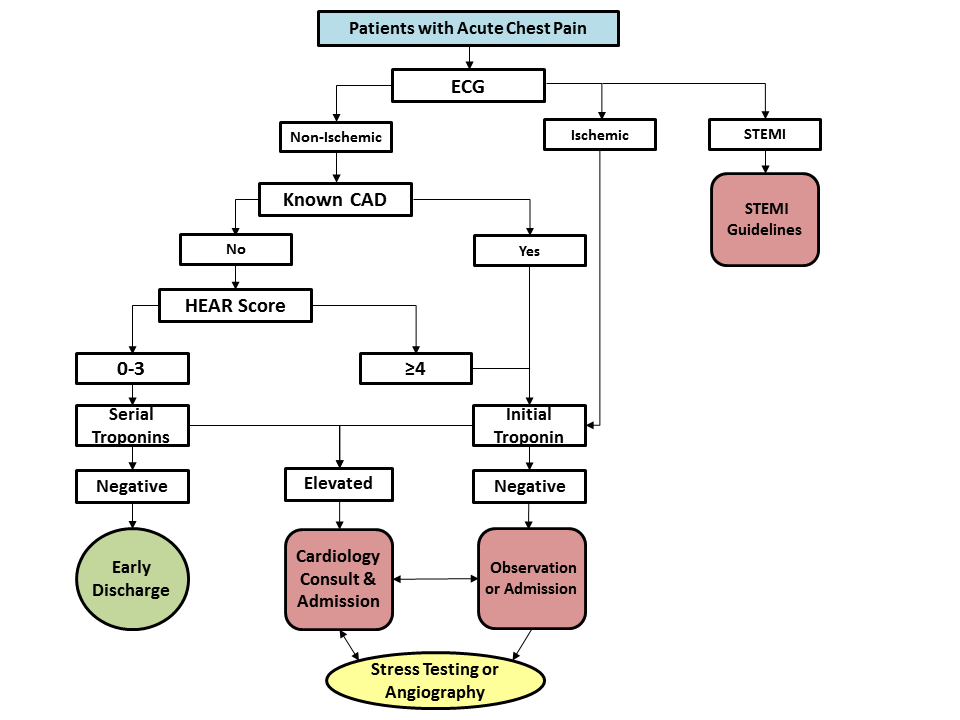


**eFigure 1** Original HEART Pathway flowchart.


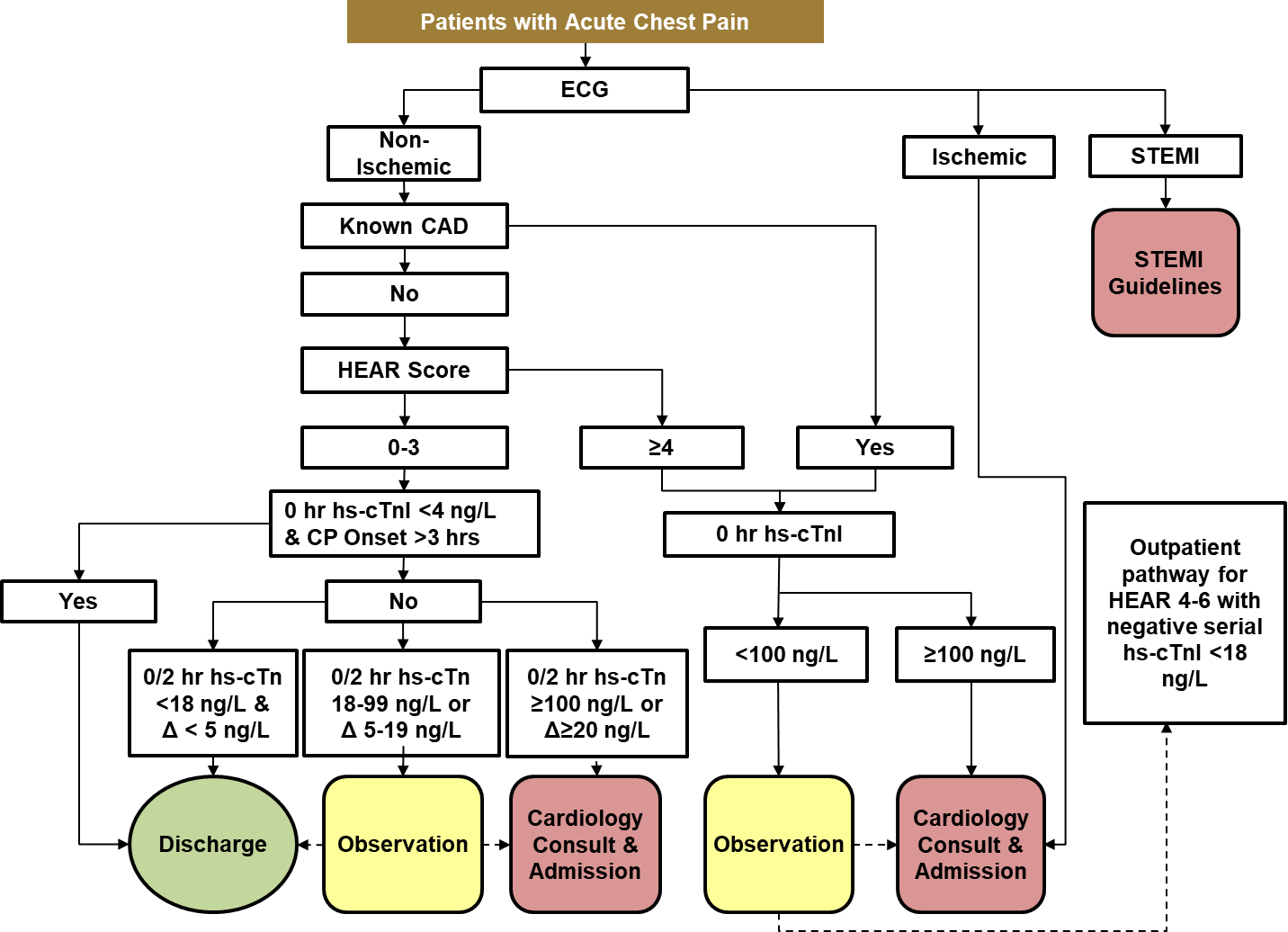


**eFigure 2:** High-sensitivity HEART Pathway (hs-HP) flowchart.

ECG – electrocardiogram, CAD – coronary artery disease, HEAR – History, ECG, Age, and Risk; hs-cTnI – high-sensitivity cardiac troponin I, CP – chest pain, STEMI – ST- Elevation Myocardial Infarction

**eTable 1a:** Difference in rates for safety and effectiveness outcomes pre- and post- high-sensitivity HEART Pathway implementation by sex.

| **Outcome** | **Female** | | | | **Male** | | | |
| --- | --- | --- | --- | --- | --- | --- | --- | --- |
|  | **Pre, N=6,460 (%)** | **Post,**  **N=7,307 (%)** | **Difference (95% CI)** | **p-value** | **Pre,**  **N=5,857 (%)** | **Post,**  **N=6,502 (%)** | **Difference (95% CI)** | **p-value** |
| **SAFETY** |  |  |  |  |  |  |  |  |
| **Index** |  |  |  |  |  |  |  |  |
| Death | 13 (0.20) | 15 (0.21) | 0.01 (-0.15 to 0.16) | >0.99 | 20 (0.34) | 31 (0.48) | 0.14 (-0.11 to 0.38) | 0.30 |
| MI | 291 (4.5) | 280 (3.8) | -0.67 (-1.4 to 0.01) | 0.05 | 529 (9.0) | 523 (8.0) | -0.99 (-2.0 to 0.02) | 0.05 |
| Revascularization | 128 (2.0) | 127 (1.7) | -0.24 (-0.71 to 0.22) | 0.32 | 316 (5.4) | 273 (4.2) | **-1.2 (-2.0 to -0.42)** | **0.002** |
| Death or MI | 300 (4.6) | 289 (4.0) | -0.69 ( -1.4 to 0.01) | 0.05 | 540 (9.2) | 545 (8.4) | -0.84 (-1.9 to 0.18) | 0.11 |
| MACE | 321 (5.0) | 308 (4.2) | **-0.75 (-1.5 to -0.04)** | **0.04** | 597 (10.2) | 586 (9.0) | **-1.2 (-2.2 to -0.12)** | **0.03** |
| **30-day Follow Up** |  |  |  |  |  |  |  |  |
| Death | 34 (0.53) | 35 (0.48) | -0.05 (-0.30 to 0.20) | 0.79 | 48 (0.82) | 48 (0.74) | -0.08 (-0.41 to 0.25) | 0.68 |
| MI | 39 (0.60) | 39 (0.53) | -0.07 (-0.34 to 0.20) | 0.67 | 64 (1.1) | 63 (1.0) | -0.12 (-0.50 to 0.25) | 0.55 |
| Revascularization | 29 (0.45) | 24 (0.33) | -0.12 (-0.34 to 0.10) | 0.32 | 70 (1.2) | 55 (0.85) | -0.35 (-0.72 to 0.02) | 0.07 |
| Death or MI | 73 (1.1) | 70 (0.96) | -0.17 (-0.53 to 0.18) | 0.36 | 108 (1.8) | 105 (1.6) | -0.23 (-0.71 to 0.25) | 0.36 |
| MACE | 82 (1.3) | 82 (1.1) | -0.15 (-0.53 to 0.23) | 0.47 | 143 (2.4) | 133 (2.0) | -0.40 (-0.94 to 0.14) | 0.15 |
| **30-day (Index + Follow-Up)** |  |  |  |  |  |  |  |  |
| Death | 47 (0.73) | 50 (0.68) | -0.04 (-0.34 to 0.25) | 0.84 | 68 (1.2) | 79 (1.2) | -0.05 (-0.35 to 0.45) | 0.85 |
| MI | 313 (4.9) | 300 (4.1) | **-0.74 (-1.5 to -0.03)** | **0.04** | 554 (9.5) | 552 (8.5) | -0.97 (-2.0 to 0.06) | 0.06 |
| Revascularization | 156 (2.4) | 149 (2.0) | -0.38 (-0.89 to 0.13) | 0.15 | 380 (6.5) | 322 (5.0) | **-1.5 (-2.4 to -0.70)** | **<0.001** |
| Death or MI | 347 (5.4) | 335 (4.6) | **-0.79 (-1.5 to -0.04)** | **0.04** | 601 (10.3) | 610 (9.4) | -0.88 (-2.0 to 0.19) | 0.11 |
| MACE | 376 (5.8) | 363 (5.0) | **-0.85 (-1.6 to -0.08)** | **0.03** | 688 (11.7) | 665 (10.2) | **-1.5 (-2.6 to -0.40)** | **0.008** |
| **EFFECTIVENESS** |  |  |  |  |  |  |  |  |
| **Index** |  |  |  |  |  |  |  |  |
| Early Discharge | 3040 (47.1) | 4924 (67.4) | **20.3 (18.7 to 22.0)** | **<0.001** | 2343 (40.0) | 3856 (59.3) | **19.3 (17.6 to 21.0)** | **<0.001** |
| Hospitalization | 2713 (42.0) | 1874 (25.7) | **-16.4 (-17.9 to -14.8)** | **<0.001** | 2922 (49.9) | 2164 (33.3) | **-16.6 (-18.3 to -14.9)** | **<0.001** |
| Objective Cardiac Testing | 1296 (20.1) | 689 (9.4) | **-10.6 (-11.8 to -9.4)** | **<0.001** | 1396 (23.8) | 924 (14.2) | **-9.6 (-11.0 to -8.2)** | **<0.001** |
| **30-day Follow Up** |  |  |  |  |  |  |  |  |
| Hospitalization | 335 (5.2) | 373 (5.1) | -0.08 (-0.84 to 0.67) | 0.86 | 452 (7.7) | 433 (6.7) | **-1.1 (-2.0 to -0.13)** | **0.03** |
| Objective Cardiac Testing | 267 (4.1) | 280 (3.8) | -0.30 (-0.97 to 0.37) | 0.39 | 275 (4.7) | 302 (4.6) | -0.05 (-0.81 to 0.71) | 0.93 |
| **30-day (Index + Follow-Up)** |  |  |  |  |  |  |  |  |
| Hospitalization | 2810 (43.5) | 2035 (27.9) | **-15.6 (-17.2 to -14.0)** | **<0.001** | 3043 (52.0) | 2304 (35.4) | **-16.5 (-18.3 to -14.8)** | **<0.001** |
| Objective Cardiac Testing | 1506 (23.3) | 928 (12.7) | **-10.6 (-11.9 to -9.3)** | **<0.001** | 1599 (27.3) | 1163 (17.9) | **-9.4 (-10.9 to -7.9)** | **<0.001** |

95% CI- 95% confidence interval, MI- myocardial infarction, MACE- major adverse cardiac event, OCT- objective cardiac testing

**eTable 1b:** Difference in rates for safety and effectiveness outcomes pre- and post- high-sensitivity HEART Pathway implementation by race.

| **Outcome** | **White** | | | | **Non-White** | | | |
| --- | --- | --- | --- | --- | --- | --- | --- | --- |
|  | **Pre, N=7,947 (%)** | **Post,**  **N=8,961 (%)** | **Difference (95% CI)** | **p-value** | **Pre, N=4,370 (%)** | **Post,**  **N=4,848 (%)** | **Difference (95% CI)** | **p-value** |
| **SAFETY** |  |  |  |  |  |  |  |  |
| **Index** |  |  |  |  |  |  |  |  |
| Death | 23 (0.29) | 38 (0.42) | 0.14 (-0.06 to 0.33) | 0.18 | 10 (0.23) | 8 (0.17) | -0.06 (-0.27 to 0.14) | 0.65 |
| MI | 605 (7.6) | 627 (7.0) | -0.62 (-1.4 to 0.18) | 0.13 | 215 (4.9) | 176 (3.6) | **-1.3 (-2.1 to -0.44)** | **0.003** |
| Revascularization | 357 (4.5) | 331 (3.7) | **-0.80 (-1.4 to -0.19)** | **0.01** | 87 (2.0) | 69 (1.4) | **-0.57 (-1.1 to -0.01)** | **0.04** |
| Death or MI | 620 (7.8) | 652 (7.3) | -0.53 (-1.3 to 0.28) | 0.21 | 220 (5.0) | 182 (3.8) | **-1.3 (-2.1 to -0.42)** | **0.003** |
| MACE | 682 (8.6) | 701 (7.8) | -0.76 (-1.6 to 0.08) | 0.08 | 236 (5.4) | 193 (4.0) | **-1.4 (-2.3 to -0.53)** | **0.001** |
| **30-day Follow Up** |  |  |  |  |  |  |  |  |
| Death | 66 (0.83) | 64 (0.71) | -0.12 (-0.39 to 0.16) | 0.44 | 16 (0.37) | 19 (0.39) | 0.03 (-0.25 to -0.30) | 0.98 |
| MI | 81 (1.0) | 79 (0.88) | -0.14 (-0.44 to 0.17) | 0.40 | 22 (0.50) | 23 (0.47) | -0.03 (-0.34 to 0.28) | 0.96 |
| Revascularization | 82 (1.0) | 63 (0.70) | **-0.33 (-0.62 to -0.04)** | **0.03** | 17 (0.39) | 16 (0.33) | -0.06 (-0.33 to 0.21) | 0.77 |
| Death or MI | 143 (1.8) | 134 (1.5) | -0.30 (-0.70 to 0.09) | 0.14 | 38 (0.87) | 41 (0.85) | -0.02 (-0.42 to 0.38) | >0.99 |
| MACE | 180 (2.3) | 167 (1.9) | -0.40 (-0.84 to 0.04) | 0.08 | 45 (1.0) | 48 (1.0) | -0.04 (-0.47 to 0.39) | 0.93 |
| **30-day (Index + Follow-Up)** |  |  |  |  |  |  |  |  |
| Death | 89 (1.1) | 102 (1.1) | 0.02 (-0.31 to 0.35) | 0.97 | 26 (0.59) | 27 (0.56) | -0.04 (-0.37 to 0.29) | 0.92 |
| MI | 641 (8.1) | 659 (7.4) | -0.71 (-1.5 to 0.11) | 0.09 | 226 (5.2) | 193 (4.0) | **-1.2 (-2.1 to -0.31)** | **0.01** |
| Revascularization | 435 (5.5) | 387 (4.3) | **-1.2 (-1.8 to -0.49)** | **<0.001** | 101 (2.3) | 84 (1.7) | -0.58 (-1.2 to 0.02) | 0.06 |
| Death or MI | 704 (8.9) | 731 (8.2) | -0.70 (-1.6 to 0.15) | 0.11 | 244 (5.6) | 214 (4.4) | **-1.2 (-2.1 to -0.25)** | **0.011** |
| MACE | 798 (10.0) | 799 (8.9) | **-1.1 (-2.0 to -0.23)** | **0.01** | 266 (6.1) | 229 (4.7) | **-1.4 (-2.3 to -0.42)** | **0.004** |
| **EFFECTIVENESS** |  |  |  |  |  |  |  |  |
| **Index** |  |  |  |  |  |  |  |  |
| Early Discharge | 3301 (41.5) | 5590 (62.4) | **20.8 (19.4 to 22.3)** | **<0.001** | 2082 (47.6) | 3190 (65.8) | **18.2 (16.1 to 20.2)** | **<0.001** |
| Hospitalization | 3857 (48.5) | 2778 (31.0) | **-17.5 (-19.0 to -16.1)** | **<0.001** | 1778 (40.7) | 1260 (26.0) | **-14.7 (-16.6 to -12.8)** | **<0.001** |
| Objective Cardiac Testing | 1842 (23.2) | 1154 (12.9) | **-10.3 (-11.5 to -9.1)** | **<0.001** | 850 (19.5) | 459 (9.5) | **-10.0 (-11.4 to -8.5)** | **<0.001** |
| **30-day Follow Up** |  |  |  |  |  |  |  |  |
| Hospitalization | 550 (6.9) | 566 (6.3) | -0.61 (-1.4 to 0.16) | 0.12 | 237 (5.4) | 240 (5.0) | -0.47 (-1.4 to 0.46) | 0.33 |
| Objective Cardiac Testing | 417 (5.2) | 430 (4.8) | -0.45 (-1.1 to 0.22) | 0.19 | 125 (2.9) | 152 (3.1) | 0.28 (-0.44 to 0.99) | 0.48 |
| **30-day (Index + Follow Up)** |  |  |  |  |  |  |  |  |
| Hospitalization | 4000 (50.3) | 2966 (33.1) | **-17.2 (-18.7 to -15.8)** | **<0.001** | 1853 (42.4) | 1373 (28.3) | **-14.1 (-16.0 to -12.1)** | **<0.001** |
| Objective Cardiac Testing | 2155 (27.1) | 1498 (16.7) | **-10.4 (-11.7 to -9.1)** | **<0.001** | 950 (21.7) | 593 (12.2) | **-9.5 (-11.1 to -8.0)** | **<0.001** |

95% CI- 95% confidence interval, MI- myocardial infarction, MACE- major adverse cardiac event, OCT- objective cardiac testing

**eTable 2.** 30-day safety and effectiveness among rule-out (one-and-done + 0/2 + non-adherent) patients with complete assessments in the post-implementation cohort by sex and race.

| **Outcomes** | **Female, N=2,752 (%)** | **Male,**  **N=2,198 (%)** | **p-value** | **Non-White,**  **N=1,921 (%)** | **White,**  **N=3,029 (%)** | **p-value** |
| --- | --- | --- | --- | --- | --- | --- |
| **SAFETY** |  |  |  |  |  |  |
| Death | 4 (0.15) | 4 (0.18) | 0.74 | 2 (0.10) | 6 (0.20) | 0.50 |
| MI | 3 (0.11) | 2 (0.09) | >0.99 | 3 (0.16) | 2 (0.07) | 0.38 |
| Revascularization | 2 (0.07) | 4 (0.18) | 0.42 | 3 (0.16) | 3 (0.10) | 0.68 |
| Death or MI | 7 (0.25) | 6 (0.27) | 0.90 | 5 (0.26) | 8 (0.26) | 0.98 |
| MACE | 8 (0.29) | 9 (0.41) | 0.48 | 7 (0.36) | 10 (0.33) | 0.84 |
| **EFFECTIVENESS** |  |  |  |  |  |  |
| Hospitalization | 212 (7.7) | 228 (10.4) | **0.001** | 163 (8.5) | 277 (9.1) | 0.43 |
| Early Discharge | 2,376 (86.3) | 1,823 (82.9) | **<0.001** | 1,628 (84.7) | 2571 (84.9) | 0.90 |
| Objective Cardiac Testing | 107 (3.9) | 112 (5.1) | **0.04** | 64 (3.3) | 155 (5.1) | **0.003** |

MI- myocardial infarction, MACE- major adverse cardiac event

**eTable 3a.** Individual components of the high-sensitivity HEART Pathway (hs-HP) in the post-implementation cohort by sex for patients with a complete hs-HP assessment (n=5055 [females], n=4648 [males]).

| **HEART Pathway Components** | **Female (%)** | **Male (%)** | **p-value** |
| --- | --- | --- | --- |
| Ischemic ECG | 73/5055 (1.4%) | 129/4648 (2.8%) | <0.001 |
| CAD | 539/4982 (10.8%) | 910/4519 (20.1%) | <0.001 |
| History |  |  | 0.002 |
| 0 | 2338/4443 (52.6%) | 2029/3609 (56.2%) |  |
| 1 | 1733/4443 (39.0%) | 1274/3609 (35.3%) |  |
| 2 | 372/4443 (8.4%) | 306/3609 (8.5%) |  |
| ECG |  |  | 0.003 |
| 0 | 2912/4443 (65.5%) | 2248/3609 (62.3%) |  |
| 1 | 1531/4443 (34.5%) | 1361/3609 (37.7%) |  |
| Age |  |  | <0.001 |
| 0 | 1545/4443 (34.8%) | 1313/3609 (36.4%) |  |
| 1 | 1794/4443 (40.4%) | 1580/3609 (43.8%) |  |
| 2 | 1104/4443 (24.8%) | 716/3609 (19.8%) |  |
| Risk |  |  | 0.049 |
| 0 | 863/4443 (19.4%) | 624/3609 (17.3%) |  |
| 1 | 2317/4443 (52.1%) | 1931/3609 (53.5%) |  |
| 2 | 1263/4443 (28.4%) | 1054/3609 (29.2%) |  |

HEART- History, ECG, Age, and Risk, and Troponin; ECG- electrocardiogram; CAD- coronary artery disease

**eTable 3b.** Individual components of the high-sensitivity HEART Pathway (hs-HP) in the post-implementation cohort by race for patients with a complete hs-HP assessment (n=3470 [non-white], n=6233 [white]).

| **HEART Pathway Components** | **Non-White (%)** | **White (%)** | **p-value** |
| --- | --- | --- | --- |
| Ischemic ECG | 51/3470 (1.5%) | 151/6233 (2.4%) | 0.002 |
| CAD | 341/3419 (10.0%) | 1108/6082 (18.2) | <0.001 |
| History |  |  | <0.001 |
| 0 | 1796/3078 (58.3%) | 2571/4974 (51.7%) |  |
| 1 | 1075/3078 (34.9%) | 1932/4974 (38.8%) |  |
| 2 | 207/3078 (6.7%) | 471/4974 (9.5%) |  |
| ECG |  |  | <0.001 |
| 0 | 1849/3078 (60.1%) | 3311/4974 (66.6%) |  |
| 1 | 1229/3078 (39.9%) | 1663/4974 (33.4%) |  |
| Age |  |  | <0.001 |
| 0 | 1220/3078 (39.6%) | 1638/4974 (32.9%) |  |
| 1 | 1334/3078 (43.3%) | 2040/4974 (41.0%) |  |
| 2 | 524/3078 (17.0%) | 1296/4974 (26.1%) |  |
| Risk |  |  | <0.001 |
| 0 | 518/3078 (16.8%) | 969/4974 (19.5%) |  |
| 1 | 1595/3078 (51.8%) | 2653/4974 (53.3.%) |  |
| 2 | 965/3078 (31.4%) | 1352/4974 (27.2%) |  |

HEART- History, ECG, Age, and Risk, and Troponin; ECG- electrocardiogram; CAD- coronary artery disease

**eTable 4a:** Sensitivity analysis of key safety and effectiveness outcomes pre- and post- high-sensitivity HEART Pathway implementation by sex for patients with complete HEART Pathway assessments.

| **Outcome** | **Female** | | | **Male** | | | **Interaction** |
| --- | --- | --- | --- | --- | --- | --- | --- |
|  | **Pre, N=2,805 (%)** | **Post,**  **N=5,055 (%)** | **Adjusted Odds Ratio (95% CI)^1^** | **Pre,**  **N=2,741 (%)** | **Post,**  **N=4,648 (%)** | **Adjusted Odds Ratio (95% CI)^1^** | **Sex x Implementation Cohort P-Value** |
| **Index** |  |  |  |  |  |  |  |
| Early Discharge | 916 (32.7) | 3,361 (66.5) | **3.93 (3.50-4.41)** | 721 (26.3) | 2,681 (57.7) | **3.53 (3.14-3.98)** | 0.10 |
| **30-day (Index + Follow-Up)** |  |  |  |  |  |  |  |
| Death or MI | 311 (11.1) | 250 (4.9) | **0.32 (0.26-0.39)** | 557 (20.3) | 461 (9.9) | **0.42 (0.36-0.49)** | 0.14 |
| MACE | 325 (11.6) | 270 (5.3) | **0.33 (0.27-0.40)** | 604 (22.0) | 506 (10.9) | **0.42 (0.36-0.49)** | 0.21 |
| Hospitalization | 1.422 (50.7) | 1,402 (27.7) | **0.33 (0.29-0.37)** | 1,689 (61.6) | 1,673 (36.0) | **0.34 (0.30-0.38)** | 0.82 |
| Objective Cardiac Testing | 874 (31.2) | 778 (15.4) | **0.38 (0.34-0.43)** | 991 (36.2) | 961 (20.7) | **0.49 (0.43-0.55)** | **0.003** |

95% CI- 95% confidence interval, MI- myocardial infarction, MACE- major adverse cardiac event, OCT- objective cardiac testing

^1^Models were adjusted for age, race, ethnicity, ED location, insurance status, smoking, prior CAD, hypertension, hyperlipidemia, and diabetes unless otherwise stated.

**eTable 4b:** Sensitivity analysis of safety and effectiveness outcomes pre- and post- high-sensitivity HEART Pathway implementation by race for patients with complete HEART Pathway assessments.

| **Outcome** | **White** | | | **Non-White** | | | **Interaction** |
| --- | --- | --- | --- | --- | --- | --- | --- |
|  | **Pre, N=3,495 (%)** | **Post,**  **N=6,233 (%)** | **Adjusted Odds Ratio (95% CI)^1^** | **Pre,**  **N=2,051 (%)** | **Post,**  **N=3,470 (%)** | **Adjusted Odds Ratio (95% CI)^1^** | **Race x Implementation Cohort P-value** |
| **Index** |  |  |  |  |  |  |  |
| Early Discharge | 1,002 (28.7) | 3,809 (61.1) | **3.81 (3.43-4.24)** | 635 (31.0) | 2,233 (64.4) | **3.63 (3.17-4.15)** | 0.75 |
| **30-day (Index + Follow-Up)** |  |  |  |  |  |  |  |
| Death or MI | 644 (18.4) | 538 (8.6) | **0.37 (0.32-0.43)** | 224 (10.9) | 173 (5.0) | **0.41 (0.32-0.52)** | 0.50 |
| MACE | 695 (19.9) | 591 (9.5) | **0.37 (0.32-0.43)** | 234 (11.4) | 185 (5.3) | **0.42 (0.33-0.53)** | 0.41 |
| Hospitalization | 2,036 (58.3) | 2,066 (33.1) | **0.33 (0.29-0.36)** | 1,075 (52.4) | 1,009 (29.1) | **0.35 (0.30-0.40)** | 0.28 |
| Objective Cardiac Testing | 1,255 (35.9) | 1,226 (19.7) | **0.45 (0.40-0.50)** | 610 (29.7) | 513 (14.8) | **0.41 (0.35-0.47)** | 0.24 |

95% CI- 95% confidence interval, MI- myocardial infarction, MACE- major adverse cardiac event, OCT- objective cardiac testing

^1^Models were adjusted for age, sex, ethnicity, ED location, insurance status, smoking, prior CAD, hypertension, hyperlipidemia, and diabetes unless otherwise stated.
